# Characterization of the circadian system in binge eating disorder and the effects of morning bright light and individually timed exogenous melatonin administration: A 4-week feasibility double-blind randomized controlled mechanistic clinical trial

**DOI:** 10.21203/rs.3.rs-10684280/v1

**Published:** 2026-08-26

**Authors:** Francisco Romo-Nava, Helen J. Burgess, Thomas Blom, Xuan Cao, Brady Williamson, Georgi Georgiev, Jakyb Stoddard, Maya Goertemoeller, John Fu, Nicole Mori, Christina Charnas, Phong Phan, Robert McNamara, Jeffrey Welge, Carlos M. Grilo, Frank Scheer, Susan L. McElroy

**Affiliations:** Lindner Center of Hope/ University of Cincinnati; University of Michigan; Yale University; Brigham and Women’s Hospital; Harvard Medical School; Lindner Center of Hope / University of Cincinnati

## Abstract

**Introduction:**

Binge eating disorder (BED) exhibits features consistent with circadian system dysregulation. This two-phase study mechanistically examined BED chronobiology. We hypothesized that dim light melatonin onset (DLMO; circadian phase) is delayed in BED and associated with illness characteristics, and be modifiable via a chronobiotic intervention.

**Methods:**

Phase 1 (P1) was a 2-week observational study comparing adults with obesity with BED (n = 43) and without BED (controls, n = 41). Phase 2 (P2) was a 4-week double-blind, randomized, controlled pilot trial in BED (NCT04724668). Participants received morning bright light (BLT; 10,000 lx) plus individually timed exogenous melatonin (3 mg) administration (BLT+ITEMA, n = 13) or sham red light (50 lx) plus placebo (n = 10). Primary outcomes were baseline DLMO differences (P1) and DLMO change (P2). Secondary/exploratory measures included wrist actigraphy and clinical features. ANCOVA, correlation, and Bayesian methods were used for statistical analysis.

**Results:**

In P1, DLMO did not differ between groups, but later DLMO correlated with greater BED severity. BED participants showed lower 24-hour locomotor amplitude and MESOR (both, posterior probability [pp] = 1), poorer diet quality, higher night eating and leptin levels (all p < 0.05). In P2, DLMO change did not differ between groups. Compared to sham+placebo, BLT+ITEMA participants had smaller reductions in peak activity (pp = 0.97) and amplitude (pp = 0.87), plus increased dietary restraint and LcN-3 fatty acids (both p < 0.05). Later baseline DLMO correlated with greater phase advance and binge eating reduction (both p < 0.05). The intervention was well tolerated.

**Conclusions:**

A later circadian phase correlated with greater BED severity, and dampened actigraphy rhythms, both support circadian disruption in BED. The mechanistic intervention is feasible and warrants refinement.

## Introduction

Binge eating disorder (BED) is characterized by recurrent episodes of eating an abnormally large amount of food in a discrete period, accompanied by a sense of loss of control, without the compensatory behaviors of bulimia nervosa^1^. It is the most prevalent eating disorder with an estimated lifetime prevalence of 1.8% globally^2–4^. BED is a significant public health problem strongly associated with increased metabolic comorbidities, higher health burden and costs^5–7^. Yet only one FDA-approved pharmacological treatment is available^8,9^. Moreover, BED shows clinical features that suggest circadian system involvement^10–15^, a robust multi-oscillator network involving the interaction between the suprachiasmatic nucleus (SCN or biological clock) and peripheral oscillators that organize and regulate physiological processes, including eating behavior^16–19^. The neurobiology of BED, including the role of the circadian system, remains poorly understood^20^, hampering the development of targeted therapeutic interventions.

Individuals with BE behavior have prominent time-of-day and circadian-related clinical features^15^, such as heightened cravings and engagement in BE behavior later in the day^15,21,22^. Cross-sectional studies show that skipping breakfast or eating less in the morning and eating more in the evening (including snacking and binge eating) are a stable trait among patients with overweight/obesity and BED^22–24^. The evening is reported as a high-risk period in which BE behavior and heightened urges to binge occur more frequently, and one third of individuals with BED report night eating^10,22^. BED adults with obesity also have a caloric intake that is lower in the morning and higher in the evening compared to individuals with obesity without BED^12^. These patterns suggest a shift in food intake timing towards the evening/night in BED with an increased risk of binge eating later in the day. Studies have also shown a relationship between self-reported late diurnal preference (i.e., evening chronotype) and BE behavior in clinical and non-clinical populations^11,25–28^. Together, these findings suggest a role of the circadian system in BED.

Actigraphy, which offers an objective method to assess aspects of the circadian system, has not been associated with psychopathological features in BED^27^, despite studies reporting actigraphy-derived sleep/wake indexes being disturbed (e.g. decreased sleep efficiency) compared to the control groups^29^. Only two studies have reported actigraphy-derived rhythm characteristics, showing decreased midline estimating statistic of rhythm (MESOR) and/or amplitude, suggesting a decreased rhythm strength in BED^13,30^. However, these results are difficult to interpret due to small sample sizes and apparent differences in experimental conditions with their control groups. Nonetheless, these preliminary findings further suggest a disrupted behavioral circadian rhythm strength in BED and require further study.

Preliminary evidence shows positive effects of chronotherapeutic interventions on BE behavior^31–34^. Bright light therapy (BLT) has shown promising therapeutic potential in a spectrum of psychiatric disorders, including depression and bipolar disorder^35^. BLT administered at 10,000-lux in the morning is thought to stimulate the endogenous wake response and entrain the circadian system^35,36^. Thus, BLT has been used to address temporal misalignment by shifting physiological circadian signaling and improving mental health outcomes^36–39^. Recent studies have used morning BLT to induce a phase advance in the treatment of BE spectrum disorders, with initial data revealing reductions in BE behavior, night eating, and food preoccupation^33,40,41^. In contrast, the use of BLT in the evening rather than the morning has not shown an effect on disordered eating, reinforcing the potential utility of morning BLT in BED^42^.

Administration of exogenous melatonin, an endogenous hormone released by the pineal gland, exerts potent phase-shifting effects in healthy individuals^43,44^ and clinical populations with circadian sleep-wake cycle disorders^45^. Melatonin has shown potential to elicit benefits in cardiometabolic outcomes, such as blood pressure and weight management, but its utility for studying BE behavior has not been characterized^46–51^. Phase response curve (PRC) studies suggest the phase shifting effects of exogenous melatonin depend on the time of administration relative to dim light melatonin onset (DLMO) of each individual^43,44^. Administration in the late afternoon/early evening can induce a phase advance towards morningness, which is enhanced when combined with morning BLT^35,52,53^. Notably, DLMO as the best-established marker of central circadian phase signaling that drives the biological transition of the brain and body from the day into the night and its clinical correlates have not been studied in BED^54–56^. Such observations open the possibility of developing mechanistic chronobiological protocols designed to induce personalized phase shifting and resynchronizing effects that enable the study of the circadian system in BED.

Here, we implemented a two-phase study involving an observational study (phase 1) with a subsequent 4-week mechanistic pilot clinical trial to evaluate the feasibility of inducing a controlled circadian phase advance (shift earlier) and realignment via a personalized chronobiological intervention (phase 2) to further characterize and evaluate the potential role of the circadian system in BED. In phase 1, we hypothesized that a delayed central circadian phase and disrupted circadian-related features (e.g., locomotor activity characteristics of rhythm) are present in BED compared to controls and that central circadian phase is associated with BED features and clinical parameters. In phase 2, we hypothesized that a personalized chronobiological intervention will induce circadian phase advance and strengthen circadian rhythms in BED is feasible, and that an earlier circadian phase at baseline and greater phase advance shifts are associated with favorable BED clinical features. The main objective of the study was thus mechanistic and not to determine the efficacy or safety of the chronobiological intervention in diagnosing or treating BED.

## Methods

### Study overview

The study consisted of two consecutive phases: the first phase involved an observational study to characterize circadian system function in adults with obesity with BED compared to a control group with obesity without BED. In the second phase, participants with BED that completed the first phase rolled over into a mechanistic pilot and feasibility clinical trial designed to evaluate the effect of a combined chronobiological intervention to induce a circadian phase advance and further evaluate the role of the circadian system in BED. The study was approved by the University of Cincinnati Institutional Review Board (UC IRB #2020 – 0345) and pre-registered at clinicaltrials.gov (NCT04724668). Study procedures involving participants were performed according to the principles outlined by the Helsinki Declaration and were conducted at the Lindner Center of Hope in Mason, Ohio.

Participants were recruited from 01/15/2021 to 05/30/2025 at the Lindner Center of Hope, the University of Cincinnati, and surrounding community through clinical referrals and advertising campaigns. Interested individuals completed an online IRB-approved prescreening questionnaire via Research Electronic Data Capture (REDCap, Vanderbilt University)^57^. Eligible candidates were contacted by the research team to complete either an IRB-approved phone screen or an inperson screening visit. Participants signed an informed consent form prior to completing any other study procedures.

### Eligibility criteria

Inclusion criteria for all participants were 18-50 years of age with a BMI ≥ 30 kg/m^2^. Inclusion criteria for the BED group were meeting DSM-5 criteria for moderate to severe BED (i.e., report at least three binge eating episodes per week during the two weeks prior to screen), confirmed via Structured Clinical Interview (SCID-5)^58^. If receiving treatment, interventions were stable (pharmacotherapy ≥ 2 months, psychotherapy ≥ 3 months) and continue at the same dose/frequency throughout the course of the study. Participants with BED could have comorbid psychiatric diagnoses if these were not the primary diagnosis or of severe intensity (e.g., mania, psychotic symptoms) according to the SCID-5. Participants on the control group were required to have no current or lifetime diagnosis of BED, bulimia nervosa, anorexia nervosa or other eating disorders, as well as no current psychiatric diagnoses (past month) according to the SCID-5. If they were receiving pharmacological or psychological interventions, stability was required for ≥ 2 months.

Exclusion criteria for all participants included current significant risk of suicide or suicidal behavior in the past year, night shift work (past month), recent (past two weeks) travel across more than one time zone, active substance use disorder, unstable medical conditions, current use of light therapy, or frequent use of melatonin (e.g., > 1/week during the past month), contraindications to study procedures or medications, and pregnancy or breastfeeding. Participants were also excluded if they used substances (e.g., caffeine or alcohol) or medication that could affect the circadian system or melatonin measurements, including Beta-blockers, pain medications (e.g., non-steroidal anti-inflammatory drugs), hypnotic sedatives, anticoagulants, anti-diabetes drugs (including GLP-1 agonists), oral corticosteroids, and other immunosuppressant medications. Active lesions or bleeding in the oral cavity that could potentially alter DLMO measurements were also exclusionary.

### Study procedures

#### Observational phase 1.

**Figure 1:**
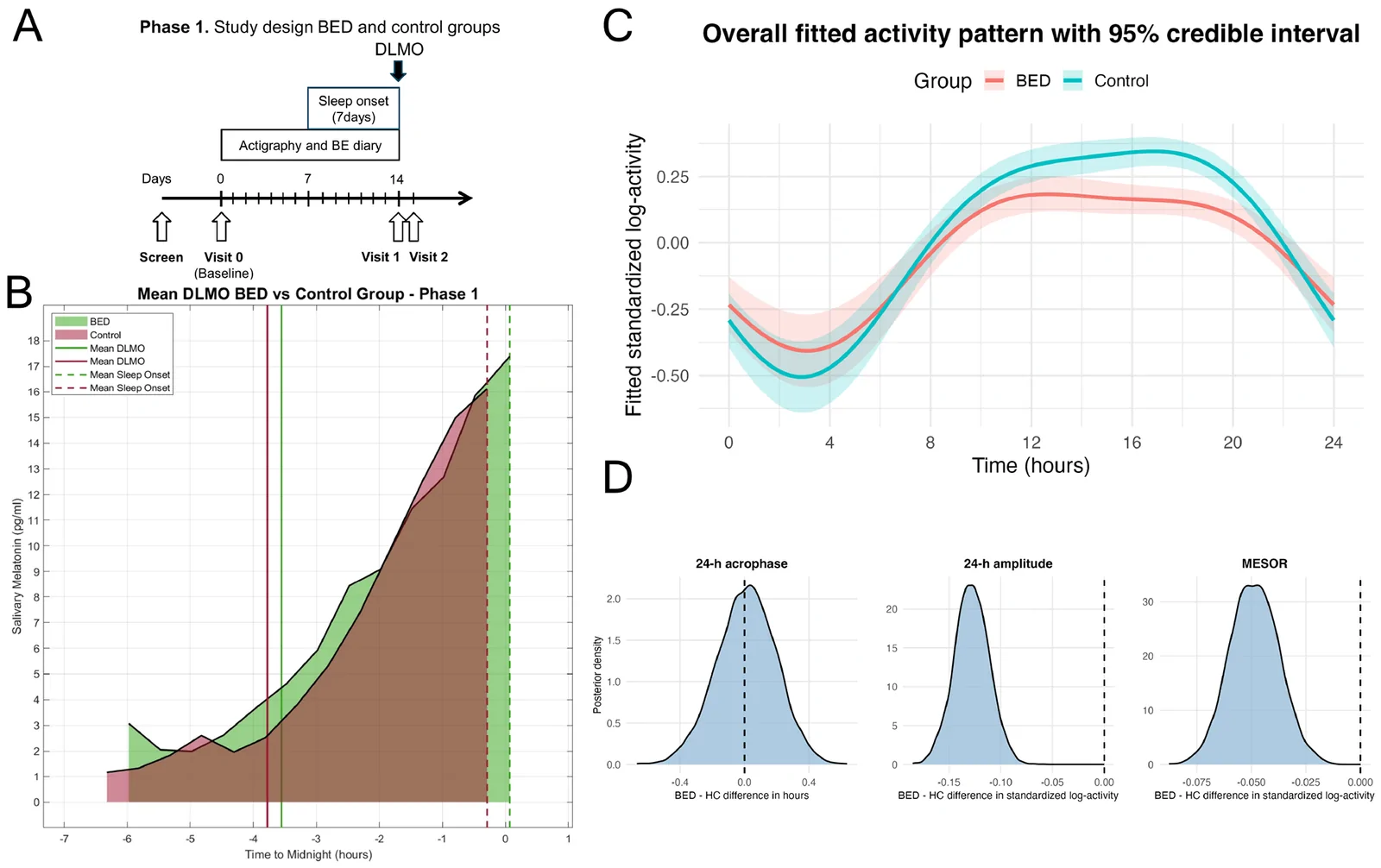
Phase 1 two-week observational study design in (**A**). Dim light melatonin onset (DLMO) including binge eating disorder (BED) and control groups (**B**). Actigraphy analyses in (C) and (D) included 57 participants (BED, n=34; Control, n=36). Panel (C) presents the cosinor-fitted locomotor activity patterns for the BED and control groups with 95% credible intervals. Panel (D) presents posterior differences in rhythm characteristics, calculated as BED minus Control. Values below zero indicate lower 24-h amplitude or MESOR, or an earlier 24-h acrophase, in the BED group relative to the control group. The x-axes show group differences in hours for acrophase and in fitted log(activity) units for amplitude and MESOR. The y-axes show posterior density. Each density curve integrates to 1; differences in peak height reflect differences in the spread of the posterior distributions.

This was a 2-week observational study (Fig. 1A). Eligible participants attended an on-site study visit and were evaluated by experienced clinicians for current and past psychiatric disorders using the SCID-5 ^58^. A medical and psychiatric history and physical exam, and safety laboratory tests were conducted. After confirmation of eligibility criteria, participants attended an initial study (P1V0) visit to begin phase 1 procedures, as recently described^59^. They were trained to complete a take home paper diary to record sleep and eating patterns^60,61^, as well as on wearing an actigraphy watch (Actiwatch Spectrum Plus, Philips, UK) on their non-dominant wrist for two weeks ^60,61^.

Participants then returned for a study visit scheduled at noon and fasting to obtain a blood sample for exploratory metabolic parameters including insulin, leptin, fibroblast growth factor-21 (FGF-21), long-chain n-3 polyunsaturated fatty acids (LcN-3 PUFAs) erythrocyte eicosapentaenoic acid (EPA) and docosahexaenoic acid (DHA) levels (EPA + DHA). Clinicians (FRN or NM) administered the Yale-Brown Obsessive Compulsive Scale modified for Binge Eating (Y-BOCS-BE) ^62^ to measure cognitive and behavioral (obsessional-compulsive) BED features and the Clinical Global ImpressionSeverity (CGI-S) to evaluate the overall clinical severity of BED^63^. The Columbia Suicide Severity Rating Scale (CSSRS)^64^ was used to evaluate suicidality. Participants also completed paper and/or electronic versions of self-report instruments to assess chronotype or diurnal preference using the morningness-eveningness questionnaire (MEQ) ^65^, sleep quality using the Pittsburgh Sleep Quality Index (PSQI)^66^, disordered eating with the brief version of the eating disorder examination questionnaire (EDE-Q 7-item version+ binge/purging items)^67^, night eating with the night eating questionnaire (NEQ)^68^, and diet quality via the Rapid Eating Assessment for Participants – Shortened Version (REAPSS)^69^. The Patient Health Questionare-9 (PHQ-9) was used to monitor depressive symptom severity^70^. Vital signs (blood pressure and heart rate) were obtained while sitting after at least 5 minutes of rest. Height, as well as weight, BMI, and body composition including fat mass, body water, lean mass, and basal metabolic rate were obtained using a body composition analyzer (MC-780U, TANITA^®^, Japan) with participants in light clothing. Blood samples were processed using ELISA assays at the Biochemistry Core Laboratory from the Schubert Research Clinic at Cincinnati Children’s Medical Center. Erythrocyte fatty acids were processed via gas chromatography at UC’s Lipidomics Research Program, as previously described ^71^.

Research staff obtained diaries and actigraphy watches from participants for their review as recently reported^59^. Briefly, diary and actigraphy data for sleep onset time (SOT) was obtained to establish DLMO saliva collection timeframe. A visual inspection allowed for corrections and verification of SOT inconsistencies between actigraphy (Actiware ^®^) and diary reports. This “verified” SOT was used to calculate a past 7-day average to establish the timing for DLMO procedures. Participants were trained on how to use a DLMO kit at home and were provided with detailed written instructions for the procedures, as recently reported in detail^59^.

After completion of DLMO training, participants went home with the instructions for saliva sample collection every 30 minutes starting 6 hours before the established SOT for a total of 13 samples. The last sample was collected at SOT, and participants kept their saliva samples frozen until they delivered them to the team, on a scheduled visit the next day^72,73^. Upon reception, procedures were reviewed by the team, and samples were sent for processing using a saliva radioimmunoassay (Novolytix, previously Buhlman) at SolidPhase (Portland, ME).

Based on previous research, the DLMO threshold was established as the average of 3 baseline melatonin concentration levels + two standard deviations (3 BL + 2 SD) ^74,75^. As recently identified by other groups conducting DLMO research, the morphology of DLMO profiles can be heterogeneous and atypical or irregular profiles are common^76^. Thus, a consensusbased approach (between FRN, HJB, and FAS) was implemented to estimate or exclude DLMO results through a systematic implementation of DLMO calculation considerations, particularly for atypical or irregular profiles (**Supplementary section 1**).

#### Intervention phase 2.

**Figure 2:**
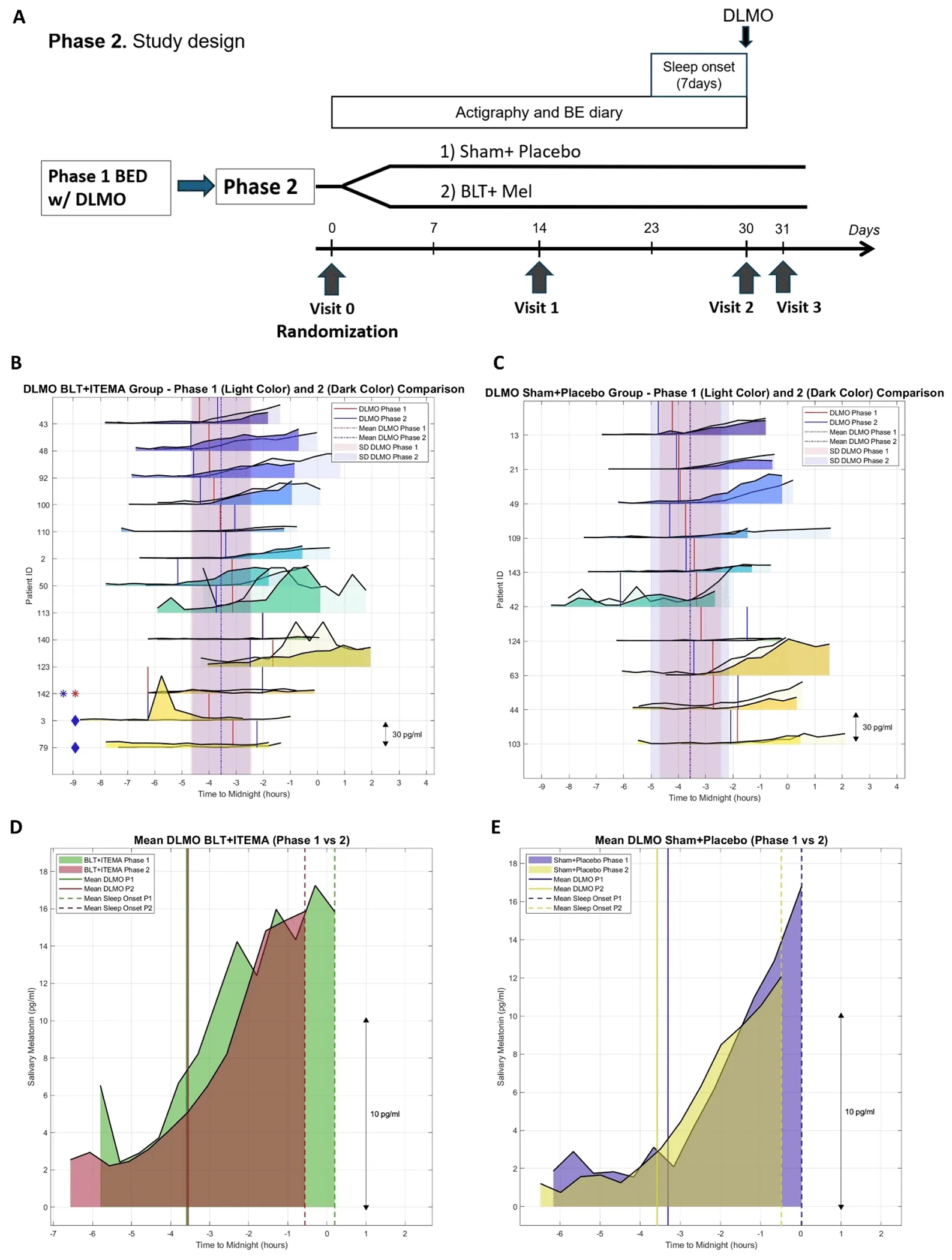
Phase 2 four-week double-blind, randomized, sham+placebo-controlled pilot clinical trial design (A). Participants with BED that successfully completed phase 1 with a dim light melatonin onset (DLMO) rolled over into phase 2 and were randomly assigned to receive bright light therapy+ Individually timed exogenous melatonin administration (BLT+ITEMA, n=11) or a sham light (dim red light at 50 luxes)+ placebo capsule (sham+placebo, n=10). Individual DLMO plots are grouped according to BLT+ITEMA in (A), or sham+placebo intervention groups (in B) and their change from phase 1 to phase 2. Blue diamond shape indicates a DLMO case removed due to unreliable or undetectable DLMO profile. A red asterisk shape indicates case excluded from phase 1 and a blue asterisk shape a case removed from phase 2 sensitivity analyses. Mean DLMO profile plots are shown for BLT+ITEMA (D) and sham+placebo groups (E). Y-axis reflects melatonin concentration (pg/ml) in saliva with each row scaled at a height of 30 pg/ml (in B and C) or 10pg/ml (in D and E). Maximum melatonin concentration levels reported by the radioimmunoassay were 50 pg/ml.

Only BED participants who completed phase 1 participated in phase 2. This phase consisted of a four-week double-blind, randomized, sham+placebo-controlled pilot and feasibility mechanistic clinical trial to further evaluate the role of the circadian system in BED (Fig. 2-A). Participants attended a study visit (P2V0) after processing phase 1 DLMO results, which were used as baseline values for phase 2. Participants with BED were randomly allocated to receive morning bright light (BLT) and individually timed exogenous melatonin administration (ITEMA) 3mg capsule 3 hours prior to phase 1 DLMO (BLT+ITEMA) or morning “sham light” sessions and a placebo capsule administered 3 hours prior to phase 1 DLMO (sham+placebo). Participants took home a light box (Northern Light Technology Boxelite OS, St-Laurent, Canada) set with 10,000 lux intensity for BLT or a “sham” version with an identical light box calibrated with 50 lux dim red light by replacing the original fluorescent tubes with a concealed dimmer and adjustable LED light bars covered with a red-light filter gel (NEEWER ^®^, Shenzhen, China). Light boxes were calibrated to provide the BLT or sham lux at 14” from the light source using a handheld light meter (REED Instruments R8140, Wilmington, NC). Research staff not involved in clinical ratings prepared, recoded, and concealed light boxes from raters to ensure successful masking^77^. Identical capsule bottles were prepared by an investigational pharmacy (Yost Pharmacy Inc., Mason, Ohio). Capsule bottles for ITEMA contained Melatonin 3mg verified through a lot certificate of analysis (donated by Pharmavite LLC, Nature Made Nutritional Products, West Hills, CA) or matching placebo capsules.

Morning light sessions lasted 30 minutes and began within 1 hour of waking. Participants used a 14” string attached to the side of the box as to maintain the correct distance and placed the light box on a stable surface (desk or table) in a comfortable, interruption-free setting. The light box was to be positioned slightly tilted downwards (at about 15 degrees) with respect to the level of the eyes. During the light sessions, they wore their actigraphy watch on a necklace, with a watch pillow, facing towards the light box as an exploratory measure of adherence. Participants were asked to close all blinds and curtains, turn off room lights, and to record the start time of each session in both their diaries and actigraphy watch. For tolerability, they were advised to glance toward but not stare directly into the light. To reduce potential melatonin-related daytime sleepiness, reduced alertness and risks while driving, study capsules could not be administered before 19:00 hrs. After training, participants started the study capsule that night and the light session the following morning.

After two weeks, participants attended a study visit (P2V1) to evaluate adverse effects and adherence. The diary and actigraphy recordings were reviewed, and clinical assessments and self-report instruments were completed. Participants were re-trained on study interventions. At day 30(+/−4 days), participants completed the last morning light session and ended study interventions. They returned for a study visit (P2V2) at noon, in fasting conditions, returned the capsule bottle and completed all clinician-administered and self-report measures and a blood draw for metabolic markers and safety laboratory tests. Adherence and adverse events were reviewed. Diary and actigraphy data were utilized to calculate past 7-day average SOT as described in phase 1. Participants were re-trained on DLMO procedures, instructed to complete them at home that night, and return with their frozen samples during a visit the next day (P2V3), where adherence was reviewed and the samples were processed to estimate DLMO.

#### Randomization and blinding.

Block randomization was used to maintain balance between groups throughout the course of the trial. Subjects were randomized in blocks of four subjects each. Within each block, two subjects were randomly assigned to each condition. Participants, study team raters, and research coordinators were blinded to study intervention. Light boxes were prepared so that BLT and sham versions were visually indistinguishable when turned off. They were placed inside identical large, zippered Oxford fabric bags (DOKEHOM ^®^, Zhejiang, China) for blinding and identified with a code. An unblinded member of the staff then re-coded the light boxes according to the randomization block order. To preserve blinding, participants were told there were different types of morning light boxes and were reminded not to disclose any characteristic of the light they received, unless previously discussed with the study clinician. Upon return, the unblinded staff member recoded the light boxes for blinded team members to sanitize, verify proper calibration, and prepare them for a new randomization block. Melatonin and placebo capsules were prepared at the investigational pharmacy in identical prescription bottles. The light box and capsule bottle were dispensed during the first study visit on phase 2.

### Outcomes

#### Phase 1.

The primary outcome measured in phase 1 was a difference in DLMO between individuals in the BED group compared to participants in the control group. Secondary outcomes included group differences in circadian parameters, including locomotor activity characteristics of rhythm and MEQ scores, and the correlations between DLMO and circadian parameters with clinical outcomes. Exploratory group comparisons were performed among metabolic parameters including serum fibroblast growth fator-21 (FGF-21), leptin, insulin, LcN-3 PUFAs, fat mass, lean mass, and total body water to evaluate preliminary metabolic mechanisms influenced by the circadian system in BED.

#### Phase 2

The primary outcome measure was the difference in DLMO change from baseline (phase 1) to phase 2 endpoint between BED individuals in the BLT+ITEMA group compared to the sham+placebo group. Secondary outcomes were phase 1 to end of phase 2 change group differences in binge eating severity, secondary circadian parameters, and the correlations between baseline DLMO and DLMO change with circadian parameters and clinical outcomes. Exploratory outcomes included metabolic parameter change and differences in self-reported questionnaires from baseline to endpoint between groups.

### Statistical analysis

Primary and secondary outcome differences between groups were analyzed using ANCOVA or logistic regression models. Given a trend level difference in age between the BED and control groups, and potential influence of age on the circadian system function^78^, age was included as a covariate in the models. Pearson correlations were used to assess the relationships among DLMO and circadian parameters with clinical outcomes. A two-sided alpha of 0.05 was used for significance testing. These analyses were conducted with SAS version 9.4 (Cary NC, USA).

Longitudinal locomotor activity data were analyzed using Bayesian mixed-effects cosinor models implemented in the brms package in R. For Phase 1 analyses, log-transformed activity was modeled using 12-hour and 24-hour harmonic terms to characterize daily activity rhythms. Activity state (Sleep vs. Rest vs. Active) was included as a time-varying covariate, and subject-specific random effects were incorporated to account for within-subject correlation and individual variability in activity rhythm patterns. Residual variance was allowed to vary across activity states. Posterior distributions of rhythm parameters were obtained from fitted hierarchical models. Subject-specific rhythm characteristics, including MESOR (average fitted activity level), amplitude, and acrophase, were derived from posterior estimates of the harmonic coefficients. Group differences were quantified using posterior contrasts derived from the fitted models.

For Phase 2 analyses, locomotor activity recordings from the final two weeks of the intervention period were analyzed using a Bayesian multi-harmonic mixed-effects waveform model incorporating 8-hour, 12-hour, and 24-hour harmonic components. This approach extends classical cosinor analysis by allowing more flexible characterization of daily activity patterns beyond a single 24-hour sinusoid^79–81^. The model included fixed effects for Phase (P1 vs. P2), Treatment (Sham+Placebo vs. BLT+ITEMA), their interactions with harmonic terms, and activity state. Subject-specific random effects were included for the 24-hour harmonic components, while residual variance was modeled as a function of activity state^82^. Rhythm characteristics were summarized directly from the fitted waveform and included MESOR, peak activity, trough activity, peak timing, trough timing, and amplitude, defined as one-half of the peak-to-trough range of the fitted waveform. Because the model incorporated multiple harmonic components (8-hour, 12-hour, and 24-hour), traditional single-component cosinor parameters are insufficient to fully characterize the resulting activity pattern. Therefore, waveform-derived measures were used to capture the overall magnitude, timing, and shape of the modeled rhythm. Longitudinal intervention (treatment) effects were evaluated using a difference-in-differences (DID) framework: DID = *(P2 − P1)*_BLT+ITEMA_ − (*P2 − P1*)_sham+Placebo_, which quantifies differential changes from baseline between treatment groups while accounting for baseline differences. Posterior means and 95% credible intervals were used to summarize parameter uncertainty.

## Results

### Phase 1.

Study enrollment flow is presented in CONSORT flow diagram for phase 1 (**Supplementary Fig. 1**). One hundred and forty-five participants were scheduled for an on-site visit, including BED (n = 80) and control (n = 65) participants. A total of 43 BED and 41 control participants completed a baseline visit. After two weeks of observational data collection, 37 BED and 41 control participants returned for a study visit. BED and control groups were similar in age, sex, race and ethnicity (Table 1).

#### Primary outcomes: DLMO group differences.

Phase 1 DLMO procedures were completed by 36 BED and 37 control participants, of which 35 in the BED group and 34 in the control group were considered to have detectable DLMOs and were included for Phase 1 analysis (**DLMO plots are included in supplementary Fig. 2**). There was no statistically significant difference between BED and control groups on phase 1 DLMO (20:27 +/−67 min vs 20:13 +/−69 min, p = 0.43) (Table 1 and Fig. 1-B). A sensitivity analysis was conducted by removing 3 BED and 2 control group DLMO profiles with atypical or irregular morphology (**supplementary Fig. 2**), which yielded similar results (20:43 +/−52 min vs. 20:22 +/− 54 min, p = 0.12).

#### Secondary and exploratory outcomes

A later baseline DLMO correlated directly with higher BED severity scores according to the Y-BOCS-BE obsessional (r = 0.37, p = 0.03) and compulsion (r = 0.51, p < 0.01) components, and the total score (r = 0.50, p < 0.01). Baseline DLMO did not correlate with BE days or BE number of episodes. A sensitivity analysis showed similar results for the correlations between baseline DLMO and the Y-BOCS-BE compulsion (r = 0.51, p = 0.01) and total scores (r = 0.46, p = 0.01), while the correlation with the obsessional component did not reach statistical significance (r = 0.31, p = 0.09) (**Supplementary Table 1**).

Circadian-related and clinical outcome differences between the BED and control groups were also evaluated. Groups were similar in SOT, DLMO calculated using 3 pg/ml as a standardized threshold, phase angle (SOT- DLMO), PSQI and MEQ scores (Table 1). The BED group showed an unhealthier diet as indicated by lower REAP-S total scores compared to the control group (25.8 +/−4.4 vs. 29.5 +/−3.9, p < 0.01). The BED group also scored higher on NEQ scores (16.9 +/− 5.4 vs. 9.4 +/− 3.6, p < 0.01) and higher rates of SCID-5 night eating syndrome (23% vs 0%, p < 0.01). Leptin levels were higher in the BED group (95.6 +/−57.5 vs. 71.6 +/−26.4, p = 0.03). There were no other group differences in metabolic markers (FGF-21, Insulin, LCn-3 PUFAs), blood pressure, heart rate, BMI, body composition parameters, or basal metabolic rate.

### Phase 1 locomotor activity characteristics of rhythm

We utilized a Bayesian framework to model locomotor actigraphy data, extract the characteristics of rhythm (acrophase, amplitude, and MESOR) and evaluate their differences across groups (Fig. 1-C). The posterior predictive checks for the model are provided in **supplementary Fig. 2**. Notably, the locomotor activity 24-hour amplitude and MESOR were decreased in the BED group compared to the control group (both with a posterior probability = 1), while 24-hour acrophase was similar between the groups (Fig. 1-D). Actigraphy 12-hour amplitude and 12-hour acrophase patterns were also similar between groups.

### Intervention Phase 2 results

A total of 29 BED participants rolled over into phase 2 and were randomly allocated to either the BLT+ITEMA (n = 15) or sham+placebo (n = 14) groups (**Phase 2 CONSORT flow diagram in supplementary Fig. 4**). A phase 2 DLMO was available for 11 participants on the BLT+ITEMA group and 10 on the sham+placebo group and were included for the analysis of the primary outcome. Complete phase 2 actigraphy data was available for 13 participants in the BLT+ITEMA group and 7 participants in the sham+placebo group. Baseline clinical and sociodemographic characteristics at baseline were similar between intervention groups (**Supplementary table 2**). According to diary entries and actigraphy watch light sensor data, morning light sessions were conducted 88% of phase 2 days, 85% of days in the BLT+ITEMA group and 93% of days in the sham+placebo group. Likewise, nightly study capsule administration adherence was documented at 92% of phase 2 days, 90% in the BLT+ITEMA group and 93% in the sham+placebo group.

#### Primary outcome: DLMO change between groups.

DLMO change from Phase 1 to the end of the phase 2 four-week intervention was similar between the BLT+ITEMA and the sham+placebo groups (0.03 +/− 1.57 hrs vs −0.27 +/−1.15 hrs, p = 0.71) (Table 2 and Fig. 2). A sensitivity analysis was conducted after removal of an atypical and irregular DLMO profile in the BLT+ITEMA group, yielding a different direction for DLMO change in the BLT+ITEMA group, but the difference with the sham+placebo group did not reach statistical significance (−0.38 +/− 0.77 hr. vs. −0.27 +/−1.15 hrs., p = 0.81). The same case was excluded in the sensitivity analysis for Phase 1 (**Supplementary Fig. 5**).

### Secondary and exploratory clinical outcomes

The BLT+ITEMA group showed a greater increase in the EDE-Q7 dietary restraint component (0.2 +/− 1.0 vs. −1.8 +/− 1.8, p < 0.01) and red blood cell LCn-3 PUFAs (0.3 +/−0.4 vs. −0.2 +/− 0.4, p < 0.01) compared to the sham+placebo group (Table 3). The BLT+ITEMA group showed a statistical trend towards a greater decrease in insulin levels (−1.8 +/−6.9 vs. 5.0 +/−9.1, p = 0.07) during the intervention phase. The intervention groups did not differ in SOT, phase angle (SOT-DLMO), BE episodes, BE days, MEQ, PSQI, REAP-S, and NEQ score change from phase 1 to the end of phase 2. There were no group differences in other EDE-Q components, Y-BOCS-BE total, obsessional, or compulsion components, or exploratory metabolic markers, BP, heart rate, BMI, body composition measures, or metabolic rate. (Table 3)

In the BLT+ITEMA group, later baseline DLMO correlated with a greater phase advance (r= −0.69, p = 0.03) and a greater decrease in BE episodes per week (r= −0.60, p = 0.03) during the intervention phase (**Supplementary table 3**). Baseline DLMO did not correlate with change in BE days or Y-BOCS-BE scores. In the sensitivity analysis, only a later baseline DLMO correlation with a greater decrease in BE episodes remained statistically significant (r= −0.71, p = 0.01). DLMO change from phase 1 to phase 2 did not correlate with BE episodes, BE days, or Y-BOCS-BE total scores, obsessional or compulsion components and the sensitivity analysis showed similar results.

In the sham+placebo group, baseline DLMO did not correlate with BE episodes, BE days, or Y-BOCS-BE total scores, obsessional or compulsion components. However, a DLMO phase advance during the intervention phase correlated with a greater decrease in Y-BOCS-BE compulsion component (r = 0.69, p = 0.04). A sensitivity analysis showed similar results. (**Supplementary table 3**).

Among participants in both groups, a later baseline DLMO correlated with a greater phase advance (r= −0.46, p = 0.04) and decrease in BE episodes (r= −0.52, p = 0.01) and trended in the same direction with greater decrease in BE days (r=−0.35, p = 0.09). In a sensitivity analysis, only the correlation between a later baseline DLMO with a greater decrease in BE episodes during the intervention phase remained statistically significant (r=−0.58, p < 0.01). (**Supplementary table 3**)

Melatonin administration time relative to DLMO did not correlate with DLMO change from phase 1 to phase 2 in any of the groups. A sensitivity analysis showed similar results. (**Supplementary table 3**)

### Phase 2 locomotor activity patterns

**Figure 3:**
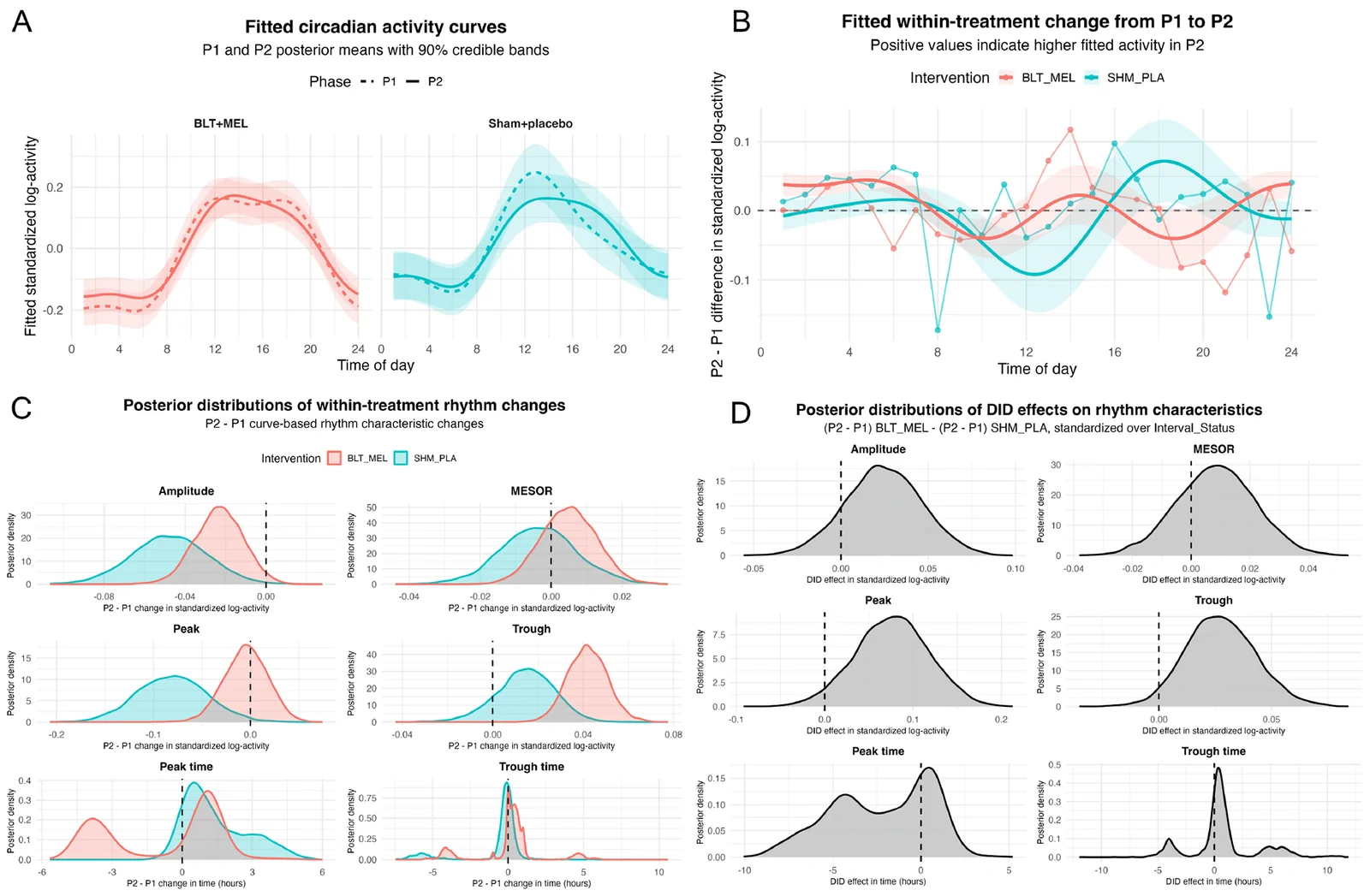
Circadian locomotor activity patterns and characteristics of rhythm in phase 2. A Bayesian framework was used to model population-level fitted circadian locomotor activity curves by phase and intervention (treatment) according to bright light + individually timed exogenous melatonin administration (BLT+ITEMA; n = 13) or sham+ placebo (n = 7) groups in (**A**). In (**B**), fitted within-intervention change curves from P1 to P2. In (**C**), posterior distributions of within-intervention characteristics of rhythm changes. In (**D**), posterior distributions of difference in difference (DID) characteristics of rhythm effects.

We examined the effects of the intervention on locomotor activity patterns and rhythm characteristics using a Bayesian modeling framework (Fig. 3A–D). Population-level fitted activity curves suggested greater changes in circadian activity patterns from P1 to P2 in the sham+placebo group compared with the BLT+ITEMA group (Fig. 3A & B). Posterior summaries of rhythm characteristics (**Supplementary tables 4 to 6**) further indicated that sham+placebo exhibited larger reductions in peak activity and waveform amplitude over time, whereas these reductions were smaller in the BLT+ITEMA group (Fig. 3C & D). The strongest evidence for intervention-related differences was observed for peak activity (posterior probability = 0.97), whereas evidence for differences in waveform amplitude was more modest (posterior probability = 0.87). In contrast, timing-related rhythm characteristics exhibited substantially greater posterior uncertainty and showed less consistent differentiation between treatment groups. Overall, these findings suggest that BLT+ITEMA may help maintain locomotor-based circadian rhythm strength relative to sham+placebo.

### Adverse events

The interventions were well tolerated, and no serious adverse events were documented. There were no differences in adverse event frequency between the BLT+ITEMA and sham+placebo groups. The most common adverse events in BLT+ITEMA and the control groups were headaches (29% vs 8%, p = 0.33), vivid dreams (21% vs. 0%, p = 0.22), and nausea (14% vs. 17%, p = 0.99). (**Supplementary Table 7**)

## Discussion

This study aimed to characterize the role of the circadian system in BED through a combined observational study phase and a mechanistic pilot and feasibility clinical trial. In the observational study, the central circadian phase was similar between the BED and control groups. However, a later circadian phase correlated with worse BED severity in the BED group. The BED group showed higher night eating syndrome rates and night eating severity, worse dietary quality patterns, and higher leptin levels, as well as decreased MESOR and amplitude in 24-hour locomotor activity circadian rhythms. During the mechanistic clinical trial phase, the intervention did not induce the expected circadian phase advance, and change in DLMO did not correlate with BE outcomes. However, a later baseline DLMO correlated with a greater phase advance and a greater decrease in BE episodes in the BLT+ITEMA group, highlighting the importance of baseline circadian phase in the response to chronobiological interventions. Compared to sham+placebo, BLT+ITEMA induced increases in self-reported dietary restraint and LcN-3 PUFAs, as well as a statistical trend towards a decrease in insulin levels, which warrant further study. Moreover, BLT+ITEMA also preserved locomotor activity rhythm strength compared to sham+placebo. The interventions were well-tolerated, and our results support their feasibility and a need for further refinement.

We utilized a home-based DLMO method to evaluate circadian phase among individuals with obesity with and without BED. While DLMO was similar between study groups, a correlation between a later DLMO and worse BE severity according to the Y-BOCS-BE supports a nuanced but strong link between central circadian phase and BE cognitive and behavioral components. Additionally, there is a decreased 24-hour rhythm amplitude and MESOR in the BED group, suggesting a diminished locomotor activity rhythm strength compared to the control group. These findings are in line with two small 5-day actigraphy studies in BED, where MESOR and or amplitude were decreased compared to a control group^13,30^. Combined, these findings support a disrupted rhythm in locomotor-activity circadian patterns in BED, and a relationship between central circadian phase and BED severity. In addition, higher night eating scores on the NEQ and higher rates of night eating syndrome in the BED group compared to the control group support their co-existence and potentially shared chronobiological mechanisms^15^ and merit further investigation.

Increased levels of leptin in the BED group are consistent with prior studies and a recent meta-analysis^83^. Increased leptin levels in BED are thought to be associated with illness-specific processes and have been proposed as trait and/or state illness marker that could be relevant for clinical monitoring and response to therapeutic interventions^83^. An unhealthier diet in BED adults with obesity compared to controls^84^, as observed in this study, and its impact on leptinemia is still a matter of study^85^. Moreover, leptin levels in healthy individuals show a clear circadian rhythm with lower levels during the day and increasing levels in the afternoon that peak at night^86^. In obesity, leptin levels are increased and its circadian rhythm dampened^87^. Whether disrupted leptin circadian rhythms play a role in the observed leptin level differences between BED and control individuals with obesity remains unknown and warrant additional study.

In this study, BLT+ITEMA did not induce a phase advance that was large enough to differentiate from the sham+placebo group. This may be largely explained by a small sample size of this feasibility and pilot study design. Moreover, heterogeneous baseline DLMO levels, and ITEMA being limited at 19hrs at the earliest, may have blunted the expected phase advance effect of the BLT+ITEMA intervention and contributed to the disparate chronobiotic effects observed across individuals. Nonetheless, BLT+ITEMA induced an increase in dietary restraint (as evaluated by the EDE-Q) but this finding should be interpreted with caution. Dietary restraint, defined broadly as the intention to restrict one’s dietary intake for the purpose of weight loss (or maintenance), has been reported as a key factor that contributes to BE behavior^88^. However, a growing body of evidence rather supports that dietary restriction (i.e., low calorie diets) or behavioral interventions aiming to decrease food-intake are associated with a decrease in BE frequency and symptoms and could represent viable intervention strategies in BED management^88^. Higher levels of flexible dietary restraint (as measured by the three-factor eating questionnaire) have predicted higher rates of binge eating abstinence and weight loss after a behavioral intervention^89^. The clinical relevance of increasing dietary restraint via BLT+ITEMA will require additional investigation.

Compared to the sham+placebo group, BLT+ITEMA induced an increase in LcN-3 PUFAs (DHA + EPA). The levels of LcN-3 PUFAS in blood are known to originate mainly from dietary intake and their increase is associated with a myriad of beneficial mental^90^ and cardiometabolic health outcomes^91^. To our knowledge, aside from LcN-3 PUFAs supplementation, there are no known non-dietary interventions that increase their levels in red blood cells. This result may indicate either a BLT+ITEMA induced shift in dietary preference/selection that was not evident from REAP-S score change or a change in fatty acid circadian metabolism^92^ through understudied pathways and exogenous melatonin effects^93,94^. This finding will also need confirmation in future studies.

Despite the stringent eligibility criteria and rigorous experimental design, our study has several limitations that should be considered when interpreting results. First, as a pilot feasibility study, a small sample size was underpowered to detect potentially small DLMO differences between the groups. Second, no adjustment was made for multiple statistical comparisons to preserve our limited power in the analysis; which should be considered when interpreting the results and their exploratory nature. Third, this study was conducted in its majority during the COVID-19 pandemic and its impact on daily routines and eating behavior is still a matter of study and could have impacted retention rates and outcomes during the study^95^. Fourth, BMI criteria did not have an upper limit, and participants had BMI levels that were on average at or above class 2 obesity, so results may not generalize to normal weight populations. It is possible that chronobiological processes involved in BED exhibit dynamic features throughout the course of illness and it may be necessary to study the chronobiology of BED across different illness stages and individuals without obesity. Fifth, even though we implemented an intense campaign to recruit male individuals, our study sample was composed mainly of female participants, and we could not examine sex differences. Sixth, our sample did not show a notably delayed DLMO which in combination with an ITEMA limit of 19 hrs. at the earliest, may have limited its potential phase advancing effect. In future studies, selecting individuals with a later DLMO at baseline may identify those more susceptible to phase advancing interventions.

Alternatively, an earlier ITEMA limit may be considered to balance potential tolerability aspects or its use in controlled settings (e.g., in-hospital). While BLT was tolerated and well accepted, participants expressed difficulties with fitting it into their daily routine and with light box portability, even with relatively short sessions. Study protocols focusing on ITEMA only or with fully portable BLT options (light glasses) and longer light sessions may offer advantages in future studies^96–98^. Seventh, as the best-established marker of central circadian phase, methods and considerations to establish DLMO thresholds warrant increased transparency as the field pursues a standardized approach that enables replicability of results. Towards that end, we offered a set of systematic decision-making considerations for DLMO determination for atypical or irregular profiles.

Overall, the experimental approach has identified a correlation between central circadian phase and BE severity, as well as a decrease in the amplitude and MESOR of actigraphy rhythms that support a role for the circadian system in BED. Moreover, our interventional phase demonstrated feasibility and its potential as a building block to develop ambulatory protocols to study the chronobiological component of BED and other psychiatric disorders. Refinement of these protocols could contribute to developing personalized chronobiological interventions that target circadian disruption in BED and other psychiatric disorders.

## Supplementary Material

This is a list of supplementary files associated with this preprint. Click to download.

- Supplementarymaterials08112026.docx

## Figures and Tables

**Table 1:** Observational phase 1 BED and control groups sociodemographic and clinical characteristics.

	Control		BED		
	(n = 37)*		(n = 35)*		
	Mean (SD)	n	Mean (SD)	n	p-value^
Age, years	42.1 (6.6)	37	38.9 (8.9)	35	0.09
Sex, # (%) female	36 (97%)	37	34 (97%)	35	0.97
Race, # (%) white	28 (76%)	37	29 (83%)	35	0.45
Ethnicity, # (%) non-Hispanic	37 (100%)	37	35 (100%)	35	NA
Sleep onset time (24 hour:min)**	23:44 (59 min)	37	24:01 (59 min)	35	0.41
DLMO BL + 2 SD (24 hour:min)**	20:13 (69 min)	34	20:27 (67 min)	35	0.43
DLMO 3 PG / ML (24 hour:min)**	20:47 (74 min)	35	21:03 (61 min)	29	0.39
Phase angle, SOT - DLMO BL, hours	3.5 (1.1)	34	3.6 (1.2)	35	0.87
PSQI global score	5.0 (2.5)	37	5.8 (2.9)	35	0.14
MEQ total score	54.3 (10.5)	37	54.7 (8.7)	35	0.70
MEQ item #19- evening type (vs. others)	9 (24%)	37	6 (17%)	35	0.44
MEQ item #19					0.82
Definitely a “morning” type	7 (19%)		7 (20%)		
Rather more a “morning” than an “evening” type	14 (38%)		9 (26%)		
Rather more an “evening” than a “morning” type	7 (19%)		13 (37%)		
Definitely “evening” type	9 (24%)		6 (17%)		
REAP-S	29.5 (3.9)	37	25.8 (4.4)	35	**< 0.01**
NEQ	9.4 (3.6)	37	16.9 (5.4)	35	**< 0.01**
Night Eating Syndrome, SCID-5	0 (0%)	37	8 (23%)	35	**< 0.01**
FGF-21	217 (217)	37	239 (179)	33	0.36
Leptin	71.6 (26.4)	37	95.6 (57.5)	34	**0.03**
Insulin	17.0 (23.4)	37	17.4 (8.8)	34	0.64
LCn-3 fatty acids (EPA + DHA)	4.3 (0.9)	37	4.0 (0.8)	34	0.23
Systolic blood pressure in mmHg	118.7 (8.2)	37	121.6 (8.8)	35	0.11
Diastolic blood pressure in mmHg	78.3 (6.1)	37	79.4 (5.3)	35	0.47
Heart rate in BPM	73.0 (8.6)	37	74.2 (9.5)	35	0.61
Body Mass Index (BMI)	39.9 (5.4)	37	42.6 (7.6)	35	0.08
Fat mass, percentage	43.1 (4.8)	37	44.2 (5.4)	35	0.28
Body water, percentage	40.7 (3.4)	37	39.9 (3.9)	35	0.28
Muscle mass, percentage	53.8 (4.6)	37	53.0 (5.3)	35	0.40
Basal metabolic rate, kcal	1948 (284)	37	2059 (363)	35	0.18

Abbreviations: Binge eating disorder (BED); Dim light melatonin onset (DLMO); Sleep onset time (SOT; Binge eating (BE); Pittsburgh Sleep Quality Inventory (PSQI); Morningness-Eveningness Questionnaire (MEQ); Rapid Eating Assessment for Participants-Short Version (REAP-S); Night Eating Questionnaire (NEQ); Eating Disorder Examination Questionnaire (EDE-Q); Yale-Brown Obsessive Compulsive Scale- adapted for Binge Eating (Y-BOCS-BE); Fibroblast Growth Factor-21 (FGF-21); Long Chain-3 fatty acids (LcN-3); Eicosapentaenoic acid and docosahexaenoic acid (EPA + DHA); beats per minute (BPM); body mass index (BMI); kilocalories (kcal).

* Sample sizes are patients with any phase I DLMO or sleep onset data

** For times after midnight, added 24 hours for mean calculation (e.g., 1 AM = 25:00)

^ controls for age on clinical variables

**Table 2:** DLMO, SOT and phase angle change between groups during the intervention phase 2.

	Sensitivity analysis*									
	BLT+ITEMA		Sham+Placebo			BLT+ITEMA		Sham+Placebo		
	Mean Change (SD)	n	Mean Change (SD)	n	p-value^	Mean Change (SD)	n	Mean Change (SD)	n	p-value^
**DLMO BL + 2 SD, hours**	0.03 (1.57)	11	−0.27 (1.15)	10	0.71	−0.38 (0.77)	10	−0.27 (1.15)	10	0.81
**SOT, hours**	−0.72 (0.74)	13	−0.33 (0.87)	10	0.28					
**Phase angle, hours**	−0.69 (1.32)	11	−0.06 (1.36)	10	0.36	−0.37 (0.85)	10	−0.06 (1.36)	10	0.56

Abbreviations: Dim light melatonin onset (DLMO); Sleep onset time (SOT).

^ controls for age

* Removed case ID 142

**Table 3:** Clinical variable change between groups during the intervention phase 2

	BED BLT+ITEMA		BED Sham+Placebo		
	Mean Change (SD)	n	Mean Change (SD)	n	p-value^
BE days/ week (last 14 days)	−2.9 (3.1)	14	−3.5 (2.5)	11	0.69
BE episodes/ week (last 14 days)	−3.2 (4.8)	14	−3.9 (2.4)	11	0.72
PSQI global score	−1.1 (2.3)	14	−0.7 (2.1)	12	0.59
MEQ total score	1.0 (5.5)	14	0.7 (5.9)	12	0.88
REAP-S	1.4 (3.6)	14	0.8 (1.7)	12	0.55
NEQ	−2.2 (5.3)	14	−3.2 (3.6)	12	0.59
EDE-Q dietary restraint	0.2 (1.0)	14	−1.8 (1.8)	12	**< 0.01**
EDE-Q shape/weight overvaluation	−0.4 (1.6)	14	−0.7 (1.8)	12	0.73
EDE-Q body dissatisfaction	−1.4 (2.9)	14	−0.5 (2.2)	12	0.40
YBOCS-BE total	−7.5 (4.8)	14	−7.8 (6.5)	12	0.92
YBOCS-BE obsessional	−2.6 (2.4)	14	−3.2 (2.8)	12	0.63
YBOCS-BE compulsion	−4.9 (3.5)	14	−4.6 (4.5)	12	0.85
FGF-21	27 (91)	13	86 (164)	9	0.31
Leptin	−5.2 (31.4)	13	2.4 (19.1)	10	0.46
Insulin	−1.8 (6.9)	13	5.0 (9.1)	10	0.07
LCn-3 fatty acids (EPA + DPA)	0.3 (0.4)	12	−0.2 (0.4)	10	**< 0.01**
Systolic blood pressure in mmHg	−4.6 (12.9)	14	−3.4 (6.9)	11	0.88
Diastolic blood pressure in mmHg	−2.7 (8.7)	14	−1.6 (4.4)	11	0.84
Heart rate in BPM	−0.4 (10.5)	14	0.1 (5.2)	11	0.99
Body Mass Index	0.3 (1.0)	14	0.4 (1.6)	11	0.80
Fat mass, percentage	0 (1.3)	14	0.1 (1.5)	11	0.87
Body water, percentage	0 (0.9)	14	−0.1 (1.2)	11	0.83
Muscle mass, percentage	0.1 (1.3)	14	−0.5 (2.4)	11	0.42
Basal metabolic rate, kcal	12.4 (54.9)	14	−6.8 (53.6)	11	0.32

Abbreviations: Dim light melatonin onset (DLMO); Sleep onset time (SOT; Binge eating (BE); Pittsburgh Sleep Quality Inventory (PSQI); Morningness-Eveningness Questionnaire (MEQ); Rapid Eating Assessment for Participants-Short Version (REAP-S); Night Eating Questionnaire (NEQ); Eating Disorder Examination Questionnaire (EDE-Q); Yale-Brown Obsessive Compulsive Scale- adapted for Binge Eating (Y-BOCS-BE); Fibroblast Growth Factor-21 (FGF-21); Long Chain-3 fatty acids (LcN-3); Eicosapentaenoic acid and docosahexaenoic acid (EPA + DHA); beats per minute (BPM); body mass index (BMI); kilocalories (kcal).

^ controls for age
